# Integrative metabolomics and hormonal profiling reveal biomarkers of milk yield efficiency in Sapera dairy goats under tropical conditions

**DOI:** 10.14202/vetworld.2025.3594-3606

**Published:** 2025-11-29

**Authors:** Rohmiyatul Islamiyati, Ismah Ulfiyah Azis, Ichlasul Amal, Muhammad Ridwan Bahar, Syahriana Sabil, Santoso Santoso, Faheem Ahmad Khan, Aeni Nurlatifah, Athhar Manabi Diansyah, Fahrul Irawan, Erni Damayanti

**Affiliations:** 1 Department of Feed and Animal Nutrition, Faculty of Animal Science, Hasanuddin University, Makassar, South Sulawesi, Indonesia; 2 Department of Animal Feed Technology, Faculty of Vocational Study, Hasanuddin University, Makassar, South Sulawesi, Indonesia; 3 Department of Animal Production, Faculty of Animal Science, Hasanuddin University, Makassar, South Sulawesi, Indonesia; 4 Department of Transfusion Medicine and Clinical Microbiology, Faculty of Allied Health Sciences, Chulalongkorn University, Bangkok, Thailand; 5 Department of Nutrition and Feed Technology, Faculty of Animal Science, Gadjah Mada University, Yogyakarta, Indonesia

**Keywords:** biomarkers, goat milk, insulin-like growth factor-1, leptin, metabolomics, precision nutrition, Sapera breed, ultra-high-performance liquid chromatography–high-resolution mass spectrometry

## Abstract

**Background and Aim:**

Milk yield variability in tropical dairy goats is driven not only by nutrition but also by complex metabolic and hormonal regulation. Conventional nutrition studies often overlook the physiological mechanisms underlying lactation efficiency. This study aimed to integrate metabolomic and hormonal analyses to identify biomarkers associated with high and low milk yield performance in Sapera goats. It provides the first untargeted ultra-high-performance liquid chromatography coupled with high-resolution mass spectrometry (UHPLC-HRMS)-based metabolomics characterization linking metabolic and endocrine profiles to lactational efficiency in tropical dairy systems.

**Materials and Methods:**

Twenty lactating Sapera goats were categorized into two groups: High-yielding (HY) (>1000 mL/day) and low-yielding (LY) (≤1000 mL/day). All animals were fed identical diets formulated according to National Research Council (2007) standards and managed under uniform housing conditions. Milk composition, blood metabolites (glucose, cholesterol, total protein, and triglycerides), and plasma hormones (leptin and insulin-like growth factor-1) were quantified using colorimetric and enzyme-linked immunosorbent assay assays. Untargeted metabolomics of milk samples was performed using UHPLC-HRMS. Metabolites were identified through multi-database annotation, and statistical comparisons were conducted using independent t-tests with p < 0.05.

**Results:**

Milk composition did not differ significantly between groups (p > 0.05). However, HY goats exhibited higher glucose and cholesterol concentrations and lower leptin levels (2.39 ± 0.42 ng/mL vs. 3.00 ± 0.44 ng/mL). Metabolomic analysis identified 213 metabolites, 19 unique to HYs, 28 to LYs, and 166 metabolites were found in both groups. HY goats showed enrichment of metabolites linked to lipid metabolism, membrane synthesis, and antioxidant defense (e.g., uric acid and phosphoserine derivatives), while LY goats displayed compounds indicative of metabolic stress and detoxification load (e.g., glycocholic acid and 3-furoic acid). Integrative correlation mapping revealed coordinated regulation between blood and milk metabolites in HY animals.

**Conclusion:**

HY and LY goats possess distinct metabolic and hormonal signatures despite similar milk composition. Identified biomarkers such as uric acid and glycerophosphoserine highlight pathways supporting efficient nutrient utilization and milk synthesis. These findings provide a foundation for precision feeding and biomarker-guided selection strategies to enhance productivity and sustainability in tropical dairy goat systems.

## INTRODUCTION

Dairy goat farming holds a strategically vital position in Indonesia’s livestock sector due to its dual economic potential and the nutritional value of goat milk. Compared with cow’s milk, goat milk is more easily digestible and rich in bioactive compounds that confer multiple health benefits, making it an important alternative for human nutrition. Despite these advantages, milk yield and quality in Indonesian dairy goats remain below optimal levels, primarily due to nutritional deficiencies, inconsistent feed availability, and seasonal fluctuations in forage quality [1]. Conventional improvement efforts have largely focused on linking dietary intake to milk yield; however, such approaches often neglect the complex physiological and metabolic processes that regulate lactation. This gap in understanding constrains progress toward improving dairy goat productivity [2]. A deeper comprehension of the metabolic and physiological mechanisms underlying milk synthesis is therefore critical to developing sustainable nutrition strategies suited for tropical dairy systems.

Recent advances in *omics* technologies, particularly metabolomics, transcriptomics, proteomics, and lipidomics, have transformed research in ruminant lactation biology by enabling comprehensive mapping of molecular pathways involved in milk production [3]. For example, metabolomic analyses using gas chromatography–time-of-flight mass spectrometry in dairy cattle have revealed intricate relationships between forage composition and systemic metabolism [4]. However, comparable multi-omics studies in goats remain limited, particularly those that integrate hormonal and metabolic indicators to inform precision nutrition strategies. The inclusion of endocrine regulators introduces a novel multi-axis framework that bridges nutritional physiology and metabolic control. Key signaling molecules, such as leptin, kisspeptin, and insulin-like growth factor-1 (IGF-1), which modulate energy metabolism and reproductive function, are particularly relevant for developing precision-nutrition models in tropical dairy goats [5]. In addition, integrated proteomic and lipidomic analyses of milk components are essential to elucidate the molecular determinants of milk synthesis efficiency.

Although dairy goat farming has become increasingly important for food security and rural economies in tropical regions, improvements in productivity remain constrained by limited mechanistic understanding of lactation biology under resource-variable conditions. Previous studies have predominantly relied on traditional nutritional assessments, focusing on diet–yield correlations, while neglecting the underlying molecular and endocrine processes that regulate nutrient utilization and milk synthesis. In ruminant science, metabolomics has emerged as a transformative tool for decoding metabolic pathways involved in lactation; however, most existing studies have concentrated on dairy cattle or temperate breeds, with limited evidence available for tropical goats, such as the *Sapera*. Furthermore, current investigations are typically unidimensional, either focusing on milk composition, metabolite profiling, or endocrine control, without integrating these physiological axes into a unified analytical framework. Consequently, the biological mechanisms that differentiate high-yielding (HY) and low-yielding (LY) goats, especially under tropical nutritional constraints, remain poorly defined [6, 7].

Another major limitation is the absence of integrated *omics* models that connect metabolite dynamics with hormonal mediators, such as leptin, kisspeptin, and IGF-1, which jointly regulate energy balance, mammary gland function, and reproductive efficiency. This disconnection between metabolic fingerprinting and endocrine regulation prevents a holistic understanding of lactational efficiency and the identification of reliable biomarkers for yield prediction and feed optimization. Moreover, most goat metabolomics studies lack translational validation, linking identified biomarkers to practical feeding interventions or breed-specific adaptive responses. As a result, the implementation of precision-nutrition programs for tropical dairy goats remains largely empirical, with limited capacity for predictive control over milk production outcomes.

To address these critical gaps, the present study represents the first untargeted ultra-high-performance liquid chromatography–high-resolution mass spectrometry (UHPLC-HRMS)-based metabolomics investigation that concurrently integrates blood biochemistry, hormonal profiling, and milk metabolome analysis in *Sapera* goats. This comprehensive approach enables the identification of yield-dependent physiological and biochemical signatures that underpin milk production. The study is structured in two sequential phases: (i) Comparative profiling to identify discriminant metabolites and hormonal markers differentiating HY and LY goats, and (ii) translation of these biomarkers into precision feeding and management frameworks tailored to tropical production systems [8].

By characterizing central metabolic nodes, such as tricarboxylic acid (TCA) cycle intermediates, lipid-derived signaling compounds, and oxidative stress markers, this framework provides a high-resolution map of nutrient partitioning mechanisms beyond conventional nutritional trials [9]. The study hypothesizes that HY goats possess distinct metabolic–endocrine adaptations that enhance energy efficiency, lipid mobilization, and antioxidant defense, thereby improving milk biosynthetic capacity under tropical stressors.

Accordingly, this study aims to (1) elucidate the integrated metabolic and hormonal landscape associated with milk yield in *Sapera* goats using UHPLC-HRMS metabolomics, (2) identify potential biomarkers predictive of lactational efficiency and nutrient utilization, and (3) develop a biomarker-driven foundation for precision nutrition and selective breeding programs suited to tropical environments [10]. Ultimately, this research is expected to catalyze a paradigm shift from empirical feeding strategies to mechanism-guided nutritional management – bridging molecular physiology with field-level productivity enhancement for sustainable dairy goat production systems.

## MATERIALS AND METHODS

### Ethical approval

All experimental procedures involving animals were approved by the Ethics Committee for the Use of Animals in Research and Learning at the Faculty of Animal Science, Hasanuddin University (Approval No. 017/UN4.12/EC/VI/2025).

### Study period and location

The study was conducted from June to August 2025. The study was conducted at the Sapera Goat Farm, Tamalate District, Jeneponto Regency, South Sulawesi, Indonesia. Milk sample preparation for testing was carried out at the Laboratory of the Faculty of Animal Science, Hasanuddin University. Milk composition testing was conducted at the SIG Laboratory, Bogor, West Java, Indonesia. Metabolomic analysis was performed at the Corpora Science Laboratory, Yogyakarta, Indonesia.

### Experimental design and criteria for grouping

A total of 20 lactating *Sapera* goats (cross between Saanen and Etawah) were selected and categorized into two experimental groups based on their average daily milk yield (n = 10 goats/group). The LY group consisted of 10 goats producing ≤1000 mL/day, whereas the HY group included 10 goats producing >1000 mL/day. At the beginning of the trial, the goats were in milk for 40 days on average, representing the early to mid-lactation stage. All animals were multiparous (2–3 parity), aged between 2.5 and 3.5 years, and had body condition scores ranging from 2.5 to 3.5.

To minimize environmental variability, all animals were maintained under uniform housing, management, and nutritional conditions. Each goat was housed individually and provided *ad libitum* access to feed and clean drinking water. The goats were fed twice daily at 07:00 and 13:00 h with a diet developed to meet the National Research Council [11] nutrient requirements for lactating dairy goats. The diet consisted of 60% forage (Napier grass) and 40% concentrate, ensuring balanced energy and protein intake across treatments. Milking was performed once daily in the morning using manual techniques to ensure consistency across animals and sampling days.

Milk yield was recorded daily throughout the 30-day experimental period, corresponding to days 30–60 of lactation, to allow adequate observation of milk production patterns under controlled conditions.

### Determination of milk composition and quality parameters

Milk yield was recorded daily for 30 consecutive days for each experimental *Sapera* doe. Manual milking was conducted once per day in the morning to ensure uniformity across animals and minimize circadian variation in milk composition.

For compositional analysis, milk samples were collected during the recording period and initially stored at 4°C before being transferred to −20°C for long-term storage until laboratory analysis. Milk components were analyzed using a Lactoscan MCCW (Milkotronic Ltd., Bulgaria).

Each sample was analyzed in duplicate, and a pooled quality control sample was measured after every ten individual samples to monitor analytical stability. The instrument was calibrated daily using standard milk references (Sigma-Aldrich, St. Louis, MO, USA). Analytical precision was expressed as the coefficient of variation (CV%), which remained below 5% for all measured components.

The following milk components were determined: total fat content, total protein, lactose, milk density, cholesterol, triglycerides, and solids-not-fat (SNF). These parameters were assessed to evaluate the relationship between milk yield and compositional quality in groups with high and low production [12].

### Blood collection and biochemical analysis

Blood metabolite testing was performed to measure glucose, cholesterol, total protein, and triglyceride levels following the procedure described by Ghavipanje *et al*. [13]. Blood samples were collected from the jugular vein in the morning before feeding and milking to minimize postprandial and circadian variations. Approximately 5 mL of blood was aseptically drawn using a 21-gauge needle and transferred into ethylenediaminetetraacetic acid-coated vacutainer tubes (BD, USA). The samples were immediately placed on ice and centrifuged at 900 x *g* for 15 min to obtain plasma.

The concentrations of glucose, cholesterol, total protein, and triglycerides were determined using commercial colorimetric assay kits (RayBiotech, Inc., USA) according to the manufacturer’s instructions. Briefly, 10 μL of plasma was mixed with 1000 μL of reagent, vortexed, and incubated at 25°C for 10 min. Absorbance was measured at 520 nm using a Genesis 10S ultraviolet visible (UV–Vis) spectrophotometer (Thermo Scientific, USA).

### Leptin and IGF-1 hormonal assays

The concentrations of plasma leptin and IGF-1 were determined using commercially available enzyme-linked immunosorbent assay kits (RayBiotech, Inc., Peachtree Corners, GA, USA), following the manufacturer’s instructions. Briefly, 100 µL of plasma or standard solution was added to each well, and the mixture was incubated for 90 min at 37°C. After washing, 100 μL of biotin-conjugated detection antibody was added, followed by horseradish peroxidase-streptavidin solution. The reaction was visualized using 3,3’,5,5’-tetramethylbenzidine (TMB) substrate and stopped with 2 N sulfuric acid. Absorbance was measured at 450 nm using a Genesis 10S UV–Vis spectrophotometer (Thermo Fisher Scientific, USA).

### Preparation of samples for metabolomics analysis

Milk samples collected from both HY and LY goat groups were aliquoted into 5 mL tubes and stored at −20°C until further analysis. An untargeted metabolomics approach using UHPLC-HRMS was employed to profile low-molecular-weight metabolites associated with milk biosynthesis and nutrient metabolism.

### Preparation of metabolomics samples

Samples stored at −20°C were thawed on ice until completely liquefied and vortexed for 10 s. Subsequently, 50 μL of milk sample and 300 μL of extraction solution (acetonitrile: methanol = 1:4, v/v) were added to a 1.5 mL microcentrifuge tube. The mixture was vortexed for 3 min and centrifuged at 12,000 rpm for 10 min at 4°C.

A volume of 200 μL of the supernatant was collected and placed in a −20°C freezer for 30 min, followed by a second centrifugation at 12,000 × *g* for 3 min at 4°C. An aliquot of 180 μL of the final supernatant was filtered and transferred for liquid chromatography–mass spectrometry (LC–MS) analysis [13].

### Integrated metabolomics workflow

This analytical configuration was optimized to generate nutrient-responsive metabolite fingerprints, enabling the identification of potential metabolomic biomarkers associated with milk biosynthesis efficiency and precision feeding in lactating goats.

Metabolomic profiling was performed using an integrated UHPLC-HRMS platform. Chromatographic separation was achieved on a Thermo Scientific Vanquish Horizon UHPLC system (Germering, Germany) equipped with a binary pump and a Hypersil GOLD analytical column (150 mm × 2.1 mm ID, 1.9 μm; Lithuania), maintained at 35°C.

The mobile phase consisted of:

- (A) 0.1% formic acid in MS-grade water
- (B) 0.1% formic acid in MS-grade acetonitrile (both Fisher Chemical, Optima LC-MS grade).

Elution was delivered at a flow rate of 0.2 mL/min under the following 24-min gradient: 2% B (0–1.5 min) → 12% B (1.5–4.5 min) → 12% B (4.5–7 min) → 24% B (7–12 min) → 48% B (12–15 min) → 60% B (15–16 min) → 100% B (16–17 min) → isocratic at 100% B (17–19 min) → return to 2% B (19–20 min) → re-equilibration (20–24 min). Injection volume was 5 μL per sample [14].

The eluates were analyzed using a Thermo Scientific Orbitrap Exploris 240 HRMS (Bremen, Germany) operating in full MS/dd-MS² mode with rapid polarity switching. Full MS scans were acquired at a resolution of 60,000 FWHM (70–1000 *m/z*, 100 ms maximum injection time, 5 ppm tolerance), and data-dependent MS² scans were recorded at 22,500 FWHM using normalized collision energies of 30, 50, and 80 eV under nitrogen gas.

Ionization was performed using an Optamax NG H-ESI (Thermo Fisher Scientific) source with spray voltages of +3500 V (positive mode) and −2500 V (negative mode). The sheath, auxiliary, and sweep gases were set to 30, 7, and 1 arbitrary units, respectively. The ion transfer tube and vaporizer temperatures were set at 275°C and 320°C, respectively.

Compared with conventional LC-MS workflows that rely on single-database annotation or unipolar scanning, the UHPLC-HRMS platform used here provided dual-polarity acquisition (±mode, 60,000 FWHM) and multi-database annotation (e.g., mzCloud, LipidMaps, KEGG, and FooDB), enhancing analytical coverage and confidence in metabolite identification. This workflow enabled nutrient-responsive metabolite fingerprinting, forming the foundation for identifying metabolomic biomarkers relevant to precision feeding [15].

### Statistical analysis

Metabolite identification and data analysis were performed using Thermo Scientific Compound Discoverer v3.3 (San Jose, CA, USA). The mzCloud (Thermo Fisher Scientific) spectral library served as the primary reference for compound identification, ensuring high-confidence matching across diverse metabolite classes, including endogenous compounds, natural products, pharmaceuticals, small molecules, steroids, hormones, and vitamins.

To ensure robust annotation, cross-validation was performed against multiple curated databases, including:

- Arita lab flavonoid structure database
- Endogenous metabolites database
- LIPID MAPS structure database (2023-01-11)
- Natural products atlas (2021_08 EFS HRAM)
- Extractables and leachables HRAM database (2023 update)
- FCCDB_2022
- ChemSpider-linked resources (ACToR, BioCyc, Bovine and Rumen Metabolome Databases, DISMA, FDA UNII, FAO, FooDB, KEGG, LIPID MAPS, Milk Composition Database, Natural Product Updates, PubChem, SDBS, Serum Metabolome Database, and related Springer Nature data).

This integrative annotation strategy adheres to current ruminant metabolomics standards and aligns with the study of Zhang *et al*. [16], which characterized the milk metabolome diversity across goats, sheep, cows, and buffaloes.

For metabolite-level group comparisons (high vs. low yield), two-sided univariate tests were performed, and p-values were corrected for multiple testing using the Benjamini–Hochberg false discovery rate. Metabolites with q < 0.05 were considered statistically significant.

For milk composition analysis, an independent-samples t-test was applied using IBM Statistical Package for the Social Sciences Statistics v21 (IBM Corp., NY, USA). Results were expressed as means ± standard deviations, and significance was determined at p < 0.05. Distinct lowercase superscript letters denote groups with significant differences.

This statistical approach ensured accurate interpretation of the biological relevance of metabolite variations in relation to milk production performance.

## RESULTS

### Milk composition stability across different yield groups

The analysis of milk composition parameters between HY and LY *Sapera* goats (Table 1) demonstrated no statistically significant differences (p > 0.05) across all measured variables, indicating general compositional stability regardless of milk yield.

**Table 1:** Stability of milk composition across different milk yield production.

Parameter	Group production		p-value

	High yield	Low yield	
Fat (%)	4.40 ± 0.24	4.41 ± 0.23	0.9187
Protein (%)	3.31 ± 0.15	3.21 ± 0.14	0.1331
Lactose (%)	4.71 ± 0.18	4.65 ± 0.18	0.4804
Solids-not-fat (%)	8.59 ± 0.23	8.65 ± 0.23	0.5681
Density (g/mL)	1.04 ± 0.01	1.03 ± 0.01	0.6105

The average fat content was 4.40 ± 0.24% in the HY group and 4.41 ± 0.23% in the LY group (p = 0.9187). Similarly, protein levels were marginally higher in high producers (3.31 ± 0.15%) than in low producers (3.21 ± 0.14%), though not significantly different (p = 0.1331). Lactose concentrations were comparable between groups (4.71 ± 0.18% in HY vs. 4.65 ± 0.18% in LY; p = 0.4804). The SNF fraction also showed minimal variation (8.59 ± 0.23% in HY vs. 8.65 ± 0.23% in LY; p = 0.5681). Likewise, milk density remained stable (1.04 ± 0.01 g/mL in HY vs. 1.03 ± 0.01 g/mL in LY; p = 0.6105). These findings collectively suggest that basic milk composition parameters are relatively unaffected by milk yield status under controlled feeding and management conditions.

### Blood metabolite profile differences between yield groups

**Figure 1:**
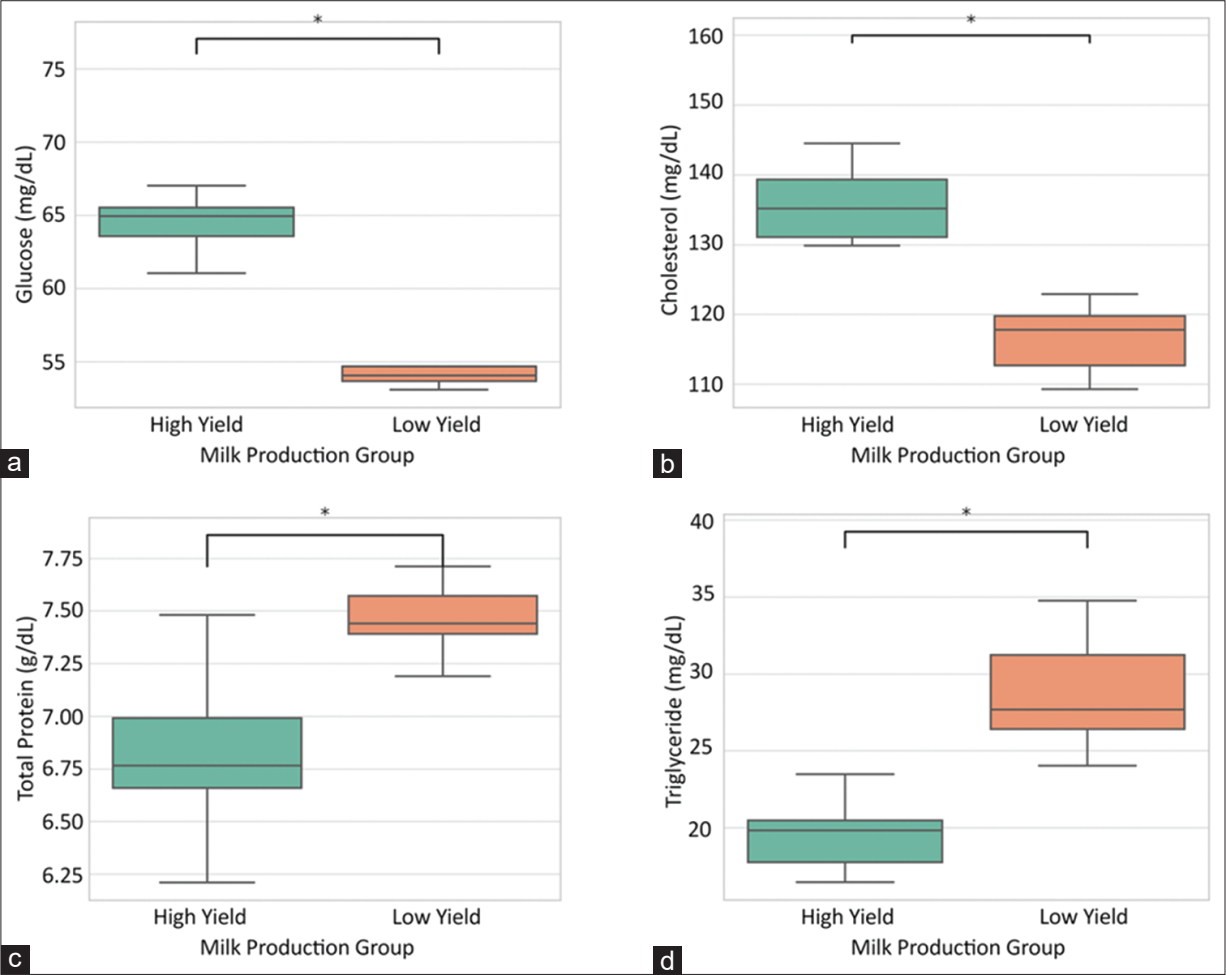
Comparison of blood metabolite concentrations between goats with high and low milk yields. Boxplots represent the distribution of (a) glucose, (b) cholesterol, (c) total protein, and (d) triglyceride levels in dairy goats grouped by milk production performance. Asterisks (*) indicate statistically significant differences between the groups (p < 0.05).

As shown in Figures 1a-d, all four blood metabolites, glucose, cholesterol, total protein, and triglycerides, exhibited statistically significant differences (p < 0.05) between the HY and LY goats. HY goats showed elevated glucose and cholesterol concentrations, whereas LY goats had higher total protein and triglyceride levels. These patterns reflect differential nutrient mobilization and energy metabolism between the two physiological states, potentially influencing milk synthesis efficiency.

### Hormonal profiles in relation to milk yield

As presented in Table 2, leptin levels differed significantly between groups (p = 0.0069), while IGF-1 levels did not show statistical variation (p = 0.1331). Leptin concentrations were significantly higher in LY goats (3.00 ± 0.44 ng/mL) than in HY goats (2.39 ± 0.42 ng/mL), suggesting a possible inverse association between leptin and milk production performance. Although IGF-1 values were not significantly different, HY goats displayed a numerically greater mean concentration (263.95 ± 11.59 ng/mL) than LY goats (239.36 ± 25.02 ng/mL), consistent with its role in mammary development and metabolic regulation.

**Table 2:** Stability of hormonal profiles across different milk yield production.

Parameter	Group production		p-value

	High yield	Low yield	
Leptin (ng/mL)	2.39 ± 0.42	3.00 ± 0.44	0.0069
Insulin-like growth factor-1 (ng/mL)	263.95 ± 11.59	239.36 ± 25.02	0.1331

### Differential metabolomic signatures across milk yield groups

**Figure 2:**
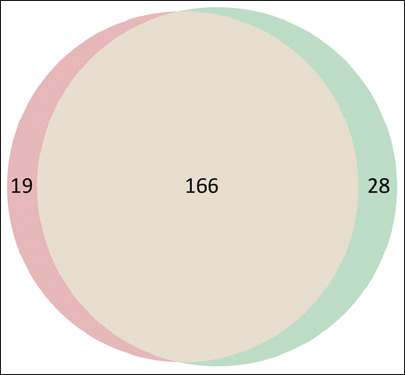
Metabolite distribution overview Left circle (pink): High yield – unique metabolites (n = 19). Right circle (green): Low yield – unique metabolites (n = 28). Overlap: Shared metabolites detected in both groups (n = 166). Totals per group: High yield = 19 + 166 = 185; Low yield = 166 + 28 = 194. Unique = Detected only in the indicated group after background filtering; shared = Detected in both groups.

Untargeted metabolomic profiling identified a total of 213 metabolites across both yield groups. Of these, 166 metabolites were shared between HY and LY goats, representing a core metabolic signature associated with general lactation physiology. Nineteen metabolites were uniquely present in the HY group and 28 were exclusive to the LY group, indicating yield-specific metabolic variation (Figure 2). These yield-dependent differences may represent key biomarkers linked to energy metabolism, lipid mobilization, and nutrient partitioning capacity, critical factors governing milk production efficiency. The identification of such unique metabolite subsets highlights the potential of metabolomics for distinguishing physiological states related to lactation and for supporting precision nutrition and selective breeding strategies in dairy goats.

### Nutritionally relevant metabolites

Based on Table 3, 47 metabolites were identified as nutritionally relevant to milk yield status. Among these, 36 were shared between the HY and LY groups, representing central metabolic pathways common to both lactational states. Four metabolites were exclusively detected in HY goats, whereas five were unique to LY goats.

**Table 3:** Differential of metabolite function to nutrient status.

Metabolite	Formula	HY	LY
(9Z)-Tetradecenoic acid	C_14_H_26_O_2_	+	+
1-(2-methoxy-octadecanyl)-sn-glycero-3-phosphoserine	C_25_H_52_NO_9_P	+	−
1-Oleoylglycerophosphocholine	C_26_H_52_NO_7_P	+	+
1-Palmitoylglycerophosphocholine	C_24_H_50_NO_7_P	+	+
1-Stearoylglycerol	C_21_H_42_O_4_	+	+
2-(Pentadecanoyl amino)-3-methylbutyric acid 2-deoxy-2-(butyrylamino)-alpha-D-glucopyranosyl ester	C_30_H_56_N_2_O_8_	−	+
2-(alpha-D-Galactosyl)-sn-glycerol3-phosphate	C_9_H_19_O_11_P	+	+
2-Amino-1,3,4-octadecanetriol	C_18_H_39_NO_3_	+	+
2-Octadecylbenzenesulfonic acid	C_24_H_42_O_3_S	+	+
2-Oxoglutaric acid	C_5_H_6_O_5_	+	+
2-[Bis (3-methylbutyl) amino]-2-(hydroxymethyl)-1,3-propanediol	C_14_H_31_NO_3_	+	+
3-Deoxyvitamin D3	C_27_H_44_	+	+
3-Furoic acid	C_5_H_4_O_3_	−	+
4-(Di-p-tolyl-amino)-benzaldehyde	C_21_H_19_NO	−	+
4-Dodecylbenzenesulfonic acid	C_18_H_30_O_3_S	+	+
4-Hexadecylbenzenesulfonic acid	C_22_H_38_O_3_S	+	+
4-Tetradecylbenzenesulfonic acid	C_20_H_34_O_3_S	+	+
4-Undecylbenzenesulfonic acid	C_17_H_28_O_3_S	+	+
4-dimethylaminobenzophenone	C_15_H_15_NO	+	+
9,10,13-trihydroxy-Octadecanoic acid	C_18_H_36_O_5_	+	+
ADENOSINE-5′-PHOSPHATE-2′,3′-CYCLIC PHOSPHATE	C_10_H_13_N_5_O_9_P_2_	+	+
Azelaic acid	C_9_H_16_O_4_	+	+
Choline	C_5_H_13_NO	+	+
Choline phosphate	C_5_H_14_NO_4_P	+	+
Citric acid	C_6_H_8_O_7_	+	+
D-Glucose 6-phosphate	C_6_H_13_O_9_P	+	+
DL-Lactic acid	C_3_H_6_O_3_	+	+
Glycocholic acid	C_26_H_43_NO_6_	−	+
Hippuric acid	C_9_H_9_NO_3_	+	+
Hydrobromic acid	HBr	+	+
Myristic acid diethanolamide	C_18_H_37_NO_3_	+	−
Methylmalonic acid	C_4_H_6_O_4_	+	+
N-(2-hydroxyethyl) hydroxy-fatty acid amide (C=6-24)	C_20_H_41_NO_3_	+	+
N-Acetyl-α-D-glucosamine 1-phosphate	C_8_H_16_NO_9_P	+	+
N~2~-(3-Carboxypropanoyl)-L-ornithyl-N-[(2S,3S,5R)-6-(butylamino)- 1-cyclohexyl-3-hydroxy-5-methyl-6-oxo-2-hexanyl]-L-valinamide	C_31_H_57_N_5_O_7_	+	+
Phthalic acid	C_8_H_6_O_4_	+	−
Pyruvic acid	C_3_H_4_O_3_	+	+
Taurochenodeoxycholic acid (sodium salt)	C_26_H_45_NO_6_S	+	+
Tributyl phosphate	C_12_H_27_O_4_P	+	+
Type IV cyanolipid ester 16:0	C_21_H_37_NO_2_	−	+
Uric acid	C_5_H_4_N_4_O_3_	+	−
Velutina laccainic acid ester (VLA)	C_33_H_54_O_5_	+	+
[FAtrihydroxy (18:0)]9_10_13-trihydroxy-11-octadecenoicacid	C_18_H_34_O_5_	+	+
sn-glycero-3-Phosphocholine	C_8_H_20_NO_6_P	+	+
Trans-aconitic acid	C_6_H_6_O_6_	+	+

+ = Presence of metabolite, − = Absence of metabolite.

### Exclusive metabolites in HY goats

As shown in Table 4, four metabolites were identified exclusively in the HY group, each associated with enhanced metabolic efficiency and milk production potential:

**Table 4:** Nutritionally relevant metabolites exclusively identified in the HY group.

Metabolite	m/z	RT (min)	Function
1-(2-methoxy-octadecanyl)- sn-glycero-3-phosphoserine	540.33038	1.856	Phosphoserine-based glycerophospholipids are involved in membrane structure and lipid metabolism in goats.
Myristic acid diethanolamide	316.28439	19.351	A fatty acid amide derivative that may interact with lipid metabolic pathways or mimic endogenous lipid-like molecules involved in membrane structure or signaling.
Phthalic acid	165.0192	9.685	An aromatic dicarboxylic acid that may be metabolized through oxidative or conjugation pathways, suggesting its potential role as a substrate in detoxification metabolism or as an indicator of systemic biochemistry affecting environmental exposure.
Uric acid	169.03545	2.496	Uric acid is a terminal product of purine degradation in ruminants, including goats, and contributes to antioxidant defense mechanisms; its presence may also reflect nucleic acid turnover or dietary protein metabolism.

- 1-(2-Methoxy-octadecanyl)-sn-glycero-3-phosphoserine: A phosphoserine-based glycerophospholipid involved in membrane structure and lipid metabolism, essential for cellular integrity during lactation
- Myristic acid diethanolamide: A lipid-like molecule that may interact with endogenous fatty acid signaling or structural lipid pathways in the mammary gland, possibly contributing to lipid transport and secretion
- Phthalic acid: An aromatic dicarboxylic acid that may participate in hepatic detoxification or serve as a marker of environmental exposure, indicating systemic metabolic resilience
- Uric acid (UA): The terminal product of purine catabolism in ruminants, functioning as an antioxidant and reflecting active nucleic acid turnover or protein metabolism supporting lactational demand.

The exclusive presence of these metabolites in HY goats suggests a distinct metabolic architecture that favors efficient lipid utilization, improved cellular function, and superior oxidative defense mechanisms.

### Exclusive metabolites in LY goats

Five metabolites were uniquely detected in the LY group (Table 5), indicative of potential metabolic stress or inefficiency that may limit milk output:

**Table 5:** Nutritionally relevant metabolites exclusively identified in the LY group.

Metabolite	m/z	RT (min)	Function
2-(Pentadecanoyl amino)-3-methylbutyric acid 2-deoxy-2-(butyrylamino)- alpha-D-glucopyranosyl este	573.41187	21.571	A potential glycolipid involved in membrane structure modulation, lipid signaling, or intracellular lipid transport; may reflect complex lipid remodeling in hepatic or adipose tissues, particularly under metabolic stress or during lactation.
3-Furoic acid	111.0087	2.55	A degradation product likely derived from furfural or lignocellulosic feed fermentation; may indicate exposure to heated feed or microbial degradation. It is processed in the liver via phase I/II xenobiotic detoxification pathways.
4-(Di-p-tolyl-amino)-benzaldehyde	302.1535	19.4	Synthetic aromatic aldehyde possibly originating from environmental contaminants or feed additives; not endogenously produced. Its detection suggests hepatic xenobiotic metabolism, particularly via cytochrome P450 enzymes.
Glycocholic acid	464.30115	17.744	Glycine-conjugated bile acid synthesized from liver cholesterol It plays a key role in emulsifying dietary fats, facilitating the absorption of lipids and fat-soluble vitamins (A, D, E, and K), and maintaining hepatic lipid homeostasis.
Type IV cyanolipid ester 16:0	336.28934	20.646	An exogenous lipid, possibly derived from cyanobacteria or algal-based feed sources. It may serve as a biomarker of dietary exposure or environmental intake. The palmitoyl moiety (C16:0) can enter the fatty acid oxidation or membrane phospholipid biosynthesis pathways.

- 2-(Pentadecanoyl amino)-3-methylbutyric acid 2-deoxy-2-(butyrylamino)-α-D-glucopyranosyl ester: A complex glycolipid possibly reflecting altered lipid remodeling or impaired lipid signaling under metabolic stress during lactation
- 3-Furoic acid: A degradation product of lignocellulosic feed fermentation, suggesting exposure to heat-treated or suboptimal feed components and activation of hepatic xenobiotic detoxification pathways
- 4-(Di-p-tolyl-amino)-benzaldehyde: A synthetic aromatic aldehyde likely derived from environmental contaminants or feed additives, indicative of cytochrome P450-mediated hepatic detoxification
- Glycocholic acid: A glycine-conjugated bile acid essential for lipid digestion and absorption; its exclusive detection may reflect altered bile acid homeostasis or compensatory mechanisms for digestive inefficiency
- Type IV cyanolipid 16:0 ester: A lipid compound potentially originating from cyanobacteria or algae-based feed ingredients, representing exogenous lipid exposure and metabolic imbalance.

Collectively, these metabolites suggest disruptions in lipid assimilation, detoxification capacity, and nutrient utilization in LY goats, which may constrain optimal lactational performance.

### Integrated correlation between blood and milk metabolites

Integrative correlation mapping between plasma and milk metabolite data revealed coherent metabolic trends across matrices. Elevated glucose and cholesterol concentrations in HY goats were positively correlated with the increased abundance of phosphoserine- and purine-derived metabolites in milk.

This coordinated regulation indicates systemic integration of energy metabolism and antioxidant defense between circulating and mammary compartments. Such dual-matrix alignment reflects a distinct physiological adaptation characteristic of HY goats, representing a novel integrative signature in caprine metabolomics.

## DISCUSSION

### Milk composition stability

This study employed an integrative metabolomics approach to elucidate the physiological and biochemical underpinnings that differentiate HY and LY *Sapera* goats in terms of milk production. Although basic milk composition parameters, including fat, protein, lactose, SNF, and density, did not differ significantly between groups (Table 1), these results are consistent with previous findings suggesting that milk quality can remain relatively stable despite yield variations [17, 18].

However, the similarity in basic composition likely masks deeper biochemical and metabolic distinctions that drive the divergence in milk output. These results suggest that production-level differences are not necessarily accompanied by proportional changes in macronutrient concentration, emphasizing the need for molecular-level investigations to explain lactational efficiency.

### Biochemical differentiation of blood profiles

A more nuanced picture emerges from the blood biochemical profiles (Figure 1), which revealed significantly higher concentrations of glucose and cholesterol in HY goats compared with LY goats. These findings are consistent with those reported by Farah *et al*. [19] and Li *et al*. [20], indicating that glucose availability is a primary limiting factor in lactogenesis due to its role as a precursor for lactose synthesis and as a critical energy substrate for the mammary gland. Elevated cholesterol levels in HY goats may further reflect enhanced steroidogenesis or lipid mobilization required to support mammary biosynthetic activity.

Conversely, higher triglyceride concentrations in LY goats might indicate impaired lipoprotein clearance or reduced lipolytic activity, possibly suggesting hepatic or systemic metabolic inefficiencies [21]. Together, these patterns imply that the biochemical signatures of HY goats are oriented toward anabolic and energy-yielding pathways, while LY goats exhibit markers of metabolic constraint or imbalance.

### Hormonal regulation and endocrine–metabolic interactions

Hormonal profiling also provided insight into the regulatory mechanisms that differentiated the two groups (Table 2). Leptin levels were significantly elevated in LY goats, which is notable considering the known inhibitory effects of leptin on feed intake and its role in energy homeostasis [22]. Elevated leptin may reflect a state of greater adiposity or reduced energy expenditure in LY goats, potentially contributing to lower nutrient partitioning toward lactation.

Although IGF-1 levels were not significantly different, the trend toward higher concentrations in HY goats aligns with its anabolic and galactopoietic roles observed in dairy cattle [23], supporting the hypothesis that IGF-1 enhances mammary epithelial proliferation and milk secretion.

The hormonal and metabolomic profiles provide complementary insights into lactational efficiency when interpreted together. The tendency for higher IGF-1 levels in HY goats coincides with metabolite patterns associated with enhanced oxidative and biosynthetic activity (e.g., phosphoserine derivatives and UA), suggesting that IGF-1 may facilitate the metabolic routing of precursors toward milk synthesis. Conversely, elevated leptin concentrations in LY goats align with metabolite signatures of energy conservation and detoxification (e.g., glycocholic acid and 3-furoic acid), implying that under suboptimal metabolic states, leptin-mediated signaling may limit nutrient flux to the mammary gland. This integrative interpretation highlights the endocrine–metabolic cross-talk that underlies yield divergence in tropical dairy goats.

### Metabolomic biomarkers of milk yield divergence

The core of the metabolic divergence between HY and LY goats was illuminated through untargeted UHPLC-HRMS metabolomics (Figure 2). Of the 213 identified metabolites, 166 were shared, representing basal lactation-related metabolism. However, 19 metabolites were unique to the HY group and 28 were unique to the LY group, reflecting functional metabolic divergence. These findings align with the concept that lactational efficiency is associated with specific biochemical adaptations in dairy cattle and sheep [24, 25].

Figure 2: Metabolite distribution overview Left circle (pink): High yield – unique metabolites (n = 19). Right circle (green): Low yield – unique metabolites (n = 28). Overlap: Shared metabolites detected in both groups (n = 166). Totals per group: High yield = 19 + 166 = 185; Low yield = 166 + 28 = 194. Unique = Detected only in the indicated group after background filtering; shared = Detected in both groups.

Four metabolites were exclusively detected in the HY group (Table 4). Among these, 1-(2-methoxy-octadecanyl)-sn-glycero-3-phosphoserine, a phosphoserine-based glycerophospholipid, plays a central role in membrane biosynthesis and lipid transport, processes essential for secretory activity in the mammary gland [26]. Although partially synthetic, myristic acid diethanolamide may mimic endogenous fatty acid amides involved in signaling or maintaining membrane integrity. Phthalic acid, typically associated with xenobiotic metabolism, may reflect enhanced hepatic detoxification capacity or tolerance to feed-derived stressors, traits that confer resilience in tropical environments. Finally, UA, a purine catabolite with antioxidant properties, may serve as a marker of nucleic acid turnover or heightened metabolic activity associated with milk synthesis [27].

In contrast, the LY group contained five unique metabolites (Table 5), many of which are indicative of metabolic stress, inefficiency, or an exogenous load. 3-Furoic acid and 4-(Di-p-tolyl-amino)-benzaldehyde are degradation products or synthetic compounds derived from heat-treated feed or environmental contaminants, implicating hepatic detoxification strain. Glycocholic acid, a bile acid, may reflect altered lipid absorption dynamics and inefficiencies in gastrointestinal lipid handling [28]. The presence of type IV cyanolipid ester further supports exposure to feed-derived exogenous lipids. Collectively, these metabolites suggest that LY goats experience greater metabolic load related to detoxification or nutrient imbalance, potentially impairing milk synthesis.

The identification of metabolite subsets specific to goats with HY and LY performance has profound implications. This indicates that metabolic plasticity and robustness, not merely nutrient intake, are critical determinants of productive efficiency. These findings reinforce earlier assertions that HY ruminants exhibit superior oxidative capacity, greater nutrient absorption efficiency, and more effective metabolic routing toward the mammary gland [29, 30]. Importantly, several HY-specific metabolites identified in this study have been reported in bovine and ovine models, strengthening the translational value of these findings [16, 25]. Beyond mechanistic insight, the metabolites identified here, particularly UA, phosphoserine derivatives, and myristic acid diethanolamide, represent promising biomarkers of lactation efficiency. Their consistent association with HY phenotypes highlights their potential utility in precision nutrition and genetic selection programs. Future research should validate these candidates using targeted metabolomics assays, such as multiple reaction monitoring in larger goat populations.

### Implications for precision nutrition and sustainable production

The ability to distinguish HY and LY phenotypes based on metabolic signatures offers a promising avenue for precision feeding, early selection, and targeted interventions in tropical dairy systems. Biomarker-informed nutritional strategies may overcome the limitations of conventional feeding approaches that rely solely on gross input–output measurements. Implementing such indicators could enable real-time dietary adjustments, reduce nutrient wastage, and improve feed conversion efficiency.

In the long-term, these biomarkers could also be incorporated into breeding programs to select for physiologically efficient, climate-resilient dairy goats. From a nutritional perspective, the identified metabolites provide actionable targets for precision diet formulation. The depletion of phosphatidylserine and UA in LY goats, for instance, suggests the need to enhance antioxidant and phospholipid synthesis through dietary supplementation with omega-3 fatty acids, Vitamin E, and selenium [31]. Similarly, altered bile acid profiles in LY goats suggest potential benefits of emulsifiers or prebiotics in improving lipid absorption.

These biomarker-driven insights can guide the design of adaptive feeding systems that align nutrient delivery with individual metabolic states, translating metabolomic signals into tangible management tools for tropical dairy production. However, this study has some limitations. The relatively small sample size may limit the generalizability of the results, and genetic background, environmental factors, or minor variations in feed intake, despite standardized conditions, may have influenced metabolic outcomes.

Future research should, therefore, expand the sample size and employ longitudinal sampling to capture dynamic changes in metabolite flux across different lactation stages. Integrating transcriptomic and rumen microbiome analyses could further elucidate how systemic and microbial metabolism interacts to regulate milk synthesis. Validation of these metabolite biomarkers under commercial field conditions will be essential for their practical deployment.

Overall, these findings underscore the value of metabolomics in decoding the physiological complexity underlying milk yield variability and highlight the transformative potential of precision nutrition in advancing sustainable tropical dairy goat production.

## CONCLUSION

This study presents the first comprehensive evaluation of biochemical, hormonal, and metabolomic factors influencing milk yield efficiency in *Sapera* dairy goats under tropical conditions. Despite the absence of significant differences in basic milk composition parameters between HY and LY groups, clear metabolic and endocrine distinctions were observed. HY goats exhibited elevated plasma glucose and cholesterol concentrations, lower leptin levels, and a metabolomic signature enriched in phosphoserine-based lipids, UA, and other metabolites associated with oxidative balance and cellular biosynthesis. In contrast, LY goats showed greater accumulation of detoxification-related compounds, such as glycocholic acid and 3-furoic acid, indicating possible metabolic stress and suboptimal nutrient utilization.

These findings reveal that milk production differences are driven not by macronutrient composition but by molecular regulation of energy metabolism, lipid turnover, and hormonal signaling. The identification of yield-specific metabolites provides a valuable foundation for developing biomarker-based precision-nutrition frameworks, enabling tailored feeding strategies that enhance feed efficiency, minimize nutrient waste, and improve lactational performance. Furthermore, these biomarkers could serve as early predictors of productive potential, facilitating genetic selection for metabolically efficient and climate-resilient dairy goats.

The principal strength of this study lies in its multi-layered analytical design integrating blood biochemistry, hormonal assays, and untargeted UHPLC-HRMS metabolomics. However, limitations include the relatively small sample size and the absence of longitudinal monitoring across lactation stages.

Future research should validate these biomarkers in larger herds under commercial conditions, incorporate transcriptomic and rumen-microbiome analyses, and assess dietary interventions targeting identified metabolic pathways. Collectively, this study underscores the promise of metabolomics as a transformative tool for advancing sustainable dairy goat production and establishes a mechanistic framework for precision nutrition in tropical livestock systems.

## DATA AVAILABILITY

All the generated data are included in the manuscript.

## AUTHORS’ CONTRIBUTIONS

RI, AMD, SaS, AN, IUA, and FI: Conceived, designed, and coordinated the study. IA, SyS, FAK, and ED: Principal investigators. RI, AMD, AN, FAK, and SaS: Designed data collection tools. AMD, IA, MRB, and IUA: Supervised field sampling, data collection, laboratory work, and data entry. RI, AMD, SyS, AN, and SaS: Statistical analysis and interpretation and drafted the manuscript. All authors have read and approved the final version of the manuscript.
